# Freshwater diatom biomonitoring through benthic kick-net metabarcoding

**DOI:** 10.1371/journal.pone.0242143

**Published:** 2020-11-18

**Authors:** Victoria Carley Maitland, Chloe Victoria Robinson, Teresita M. Porter, Mehrdad Hajibabaei

**Affiliations:** Centre for Biodiversity Genomics & Department of Integrative Biology, University of Guelph, Guelph, Ontario, Canada; University of Hyogo, JAPAN

## Abstract

Biomonitoring is an essential tool for assessing ecological conditions and informing management strategies. The application of DNA metabarcoding and high throughput sequencing has improved data quantity and resolution for biomonitoring of taxa such as macroinvertebrates, yet, there remains the need to optimise these methods for other taxonomic groups. Diatoms have a longstanding history in freshwater biomonitoring as bioindicators of water quality status. However, multi-substrate periphyton collection, a common diatom sampling practice, is time-consuming and thus costly in terms of labour. This study examined whether the benthic kick-net technique used for macroinvertebrate biomonitoring could be applied to bulk-sample diatoms for metabarcoding. To test this approach, we collected samples using both conventional multi-substrate microhabitat periphyton collections and bulk-tissue kick-net methodologies in parallel from replicated sites with different habitat status (good/fair). We found there was no significant difference in community assemblages between conventional periphyton collection and kick-net methodologies or site status, but there was significant difference between diatom communities depending on site (*P* = 0.042). These results show the diatom taxonomic coverage achieved through DNA metabarcoding of kick-net is suitable for ecological biomonitoring applications. The shift to a more robust sampling approach and capturing diatoms as well as macroinvertebrates in a single sampling event has the potential to significantly improve efficiency of biomonitoring programmes that currently only use the kick-net technique to sample macroinvertebrates.

## Introduction

As climate change and other anthropogenic impacts continue to alter the environment, there is an increasing need for comprehensive ecological assessment. Rapid and robust biomonitoring is essential for informing management plans and mitigating further environmental degradation [1–3]. Freshwater biomonitoring typically involves sampling a range of aquatic taxa, with particular focus on biological indicator taxa, to assess environmental conditions based on diversity, richness, structure and function of the existing communities [3–5].

Traditionally, biomonitoring data is generated through morphological taxonomic classifications, however there has been a recent shift towards DNA-based identification using metabarcoding [6] coupled with high throughput sequencing [7]. In aquatic systems such as wadable streams, a combination of bulk-tissue benthic sampling using kick-net methodology with DNA metabarcoding, facilitates rapid data collection whilst maintaining data integrity [8–10]. The metabarcoding approach has been employed for numerous biomonitoring studies involving macroinvertebrates [11, 12] for assessing freshwater health [5, 10, 13].

In addition to benthic macroinvertebrates, diatoms (members of Bacillariophyta) are also ideal biomonitoring target taxa for assessing freshwater system conditions [14–17]. These single-celled algae have a short generation time which allows for rapid responses to physical, chemical and biological changes in the environment [14, 15, 18]. Similar to macroinvertebrates, diatoms are ubiquitous and are used to create biotic indices that can accurately report freshwater quality [16, 19, 20]. Studies have shown that diatoms respond more readily to the presence of heavy metal pollutants compared to macroinvertebrates, which are generally more sensitive to shifts in hydrological conditions [18, 21–23]. Monitoring only one of these taxonomic groups to assess overall ecosystem health could potentially cause gaps in knowledge that could subvert subsequent management strategies. Hence, diatoms are being used in a number of national and regional biomonitoring programmes.

Current field methods for sampling periphyton (a combination of algae, cyanobacteria, microbes, and detritus) for river ecological assessments (i.e. rock-scraping in addition to microhabitat sampling), can be time-consuming and laborious, depending on the number of biological replicates collected and microhabitats present at each site [24–26]. The basic level of diatom collection involves rock scraping of three to five stones per site [27], which has previously been utilised in number of studies [27–29]. However, taking this sole approach often fails to detect the true diversity of diatoms, especially concerning planktonic diatom species [24–26, 30, 31]. Across the world, specific communities and species of diatoms are known to have different substrate and water column preferences [32–34], highlighting the importance of sampling microhabitats in addition to rock scraping for estimating true diatom diversity.

In terms of laboratory processing, after collection, diatom samples are typically fixed and visualised using light microscopy [35–38]. For identification, microscopy standards are followed [37–39] for classification to species or genus level. Within recent years, there has been an increase in applying DNA metabarcoding to identify diatom communities [15, 16, 40, 41]. This involves the manual homogenized of periphyton scrapings into single samples, which are then processed via standard diatom metabarcoding procedures [42, 43]. Alternative sampling methods, such as collection through the benthic kick-net technique, have not been tested for diatom biomonitoring applicability, however it is expected that this stand alone technique would drastically reduce time spent collecting samples. The ability to study diatom and macroinvertebrate assemblages from a single sample would allow biomonitoring programs to achieve an intensive appraisal of freshwater conditions. In a rapidly changing world, streamlining current methodology to obtain as much data in as little time as possible is crucial.

Because DNA-based analysis of environmental samples such as contents of a kick-net sample can provide a broad spectrum of organisms in the habitat sampled, we hypothesized that kick-net metabarcoding will provide diatom biodiversity comparable to commonly used multi-substrate periphyton collection method. Specifically, we aimed to 1) investigate the feasibility of kick-net sampling for capturing community assemblages of freshwater diatoms versus conventional periphyton collection using a high throughput sequencing coupled metabarcoding approach and 2) compare diatom community assemblages across a known habitat quality scale (Good and Fair) using both conventional and kick-net sampling to investigate presence of diatom indicator groups.

## Methods

### Field sampling

Samples were collected in November 2019 from Grand River tributaries across four study sites in Waterloo, Ontario (S1 Table in S1 File). No specific permissions were required for sampling these sites because they are on public land and the field studies did not involve endangered or protected species. Status and location data were provided by Dougan & Associates based on a 2018 benthos biomonitoring project for the City of Waterloo (S1 Table in S1 File). The four selected sites were a subset of the sites from this project and were chosen based on accessibility and habitat quality. Hilsenhoff Biotic Index ranges (weighted by species) informed the habitat quality scale [44] which categorized sites into ‘Good’ (4.51–5.50) and ‘Fair’ (5.51–6.50).

**Fig 1:**
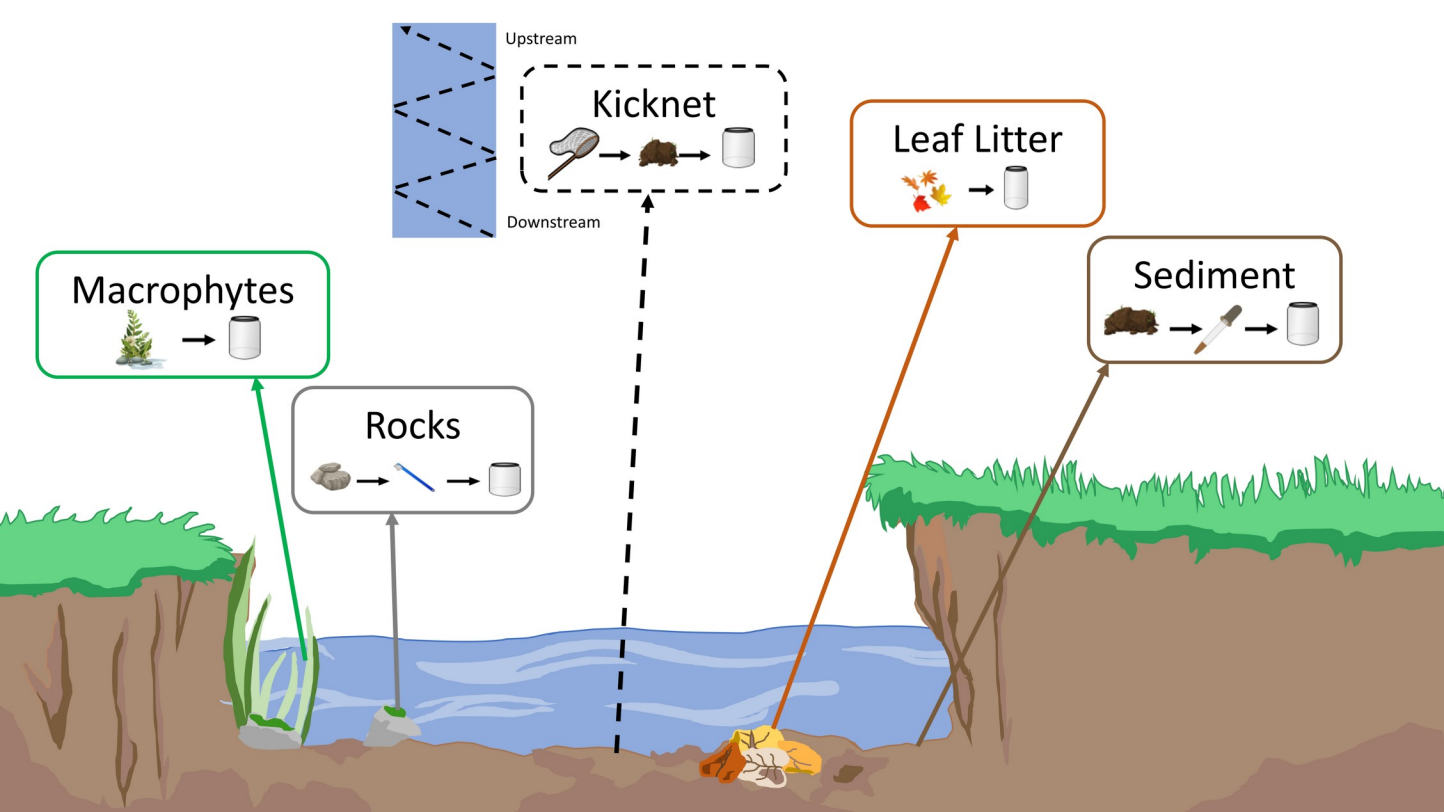
Sampling schematic of periphyton collection. A benthic kick-net sample (dotted line) was collected first by kicking benthic sediment into a D-net, from downstream to upstream in a zig-zag across the sampling reach. Microhabitats (rocks, leaf littler, macrophytes and sediment) from the same reach were sampled second for periphyton collection following previously published, standardised protocols [24].

Collection occurred in riffles, starting with a benthic kick-net sample, followed by subsequent periphyton collection of microhabitats representative of the reach (S2 Table in S1 File; Fig 1). Periphyton collection refers to the sampling of sediment, rock, macrophytes and leaf litter for diatoms. Three replicates of each sampling type were collected at each site. Kick-net collection followed the Canadian Aquatic Biomonitoring Network [CABIN] protocol [45]. Effort was standardized to three minutes. The sampler moved up stream in a zig-zag pattern to encompass all microhabitats within the reach. Periphyton collection samples were comprised of three specimens per microhabitat type to account for variability within the microhabitat [24]. Negative controls, consisting of molecular grade water, were collected prior to the collection of each rock sample (n = 9) to ensure the toothbrushes used for scraping biofilms from rocks had been adequately sterilised (microbe and DNA-free) (S3 Table in S1 File). All other samples were collected using manufacture-sealed sterile equipment. All samples were collected in 1L sample jars and placed in a cooler to transport back to the lab. Upon arrival at the lab, samples (n = 45) were preserved using 100% ethanol and stored in a -20°C freezer until processing.

### Sample validation and extraction

To account for potential false negatives [46], diatom presence in the samples was confirmed using microscopy. A small amount of ethanol used to preserve the samples was placed on a slide and observed under a compound microscope at 100X magnification. Visual inspection confirmed the presence of diatoms in each sample type (S1 Fig in S1 File), however no taxonomic information was taken as morphological identification was beyond the scope of this study.

Once diatom presence was validated, all samples were homogenized using standard blenders decontaminated by washing with ELIMINase® (VWR, Canada) then rinsing with deionized water before treating with UV light for 30 minutes. Homogenate was subsequently transferred to 50 mL Falcon tubes, where one tube was set aside and centrifuged at 2400 rpm for two minutes. Supernatant was removed and residual pellets were incubated at 70°C until fully dried. Next, approximately 300 mg dried tissue was subsampled into PowerBead tubes and DNA extractions were completed using the DNeasy Power Soil kit (Qiagen, CA) following the manufacturer’s protocol. The only exception being that 50 μL of buffer C6 (TE) was used for final elution. Negative controls containing no tissue were also included with each batch of extractions. All negative controls failed to amplify and therefore were not sequenced.

### DNA amplification, library preparation and sequencing

Amplification targeted the 312 base pair long region of the chloroplast gene ribulose bisphosphate carboxylase large chain (rbcL) using five diatom specific primers. Following the methods of Rivera et al. [15], forward primers Diat_rbcL_708F_1 (5’-AGGTGAAG- TAAAAGGTTCWTACTTAAA-3’), Diat_rbcL_708F_2 (5’-AGGT- GAAGTTAAAGGTTCWTAYTTAAA-3’) and Diat_rbcL_708F_3 (5’-AGGTGAAAC- TAAAGGTTCWTACTTAAA-3’) were combined in an equimolar mix. Two reverse primers, Diat_rbcL_R3_1 (5’-CCTTCTAATTTACC- WACWACTG-3’) and Diat_rbcL_R3_2 (5’-CCTTCTAATTTACCWA-CAACAG-3’), were also combined and used for amplification. Each reaction used the following reagents: 17.5 μL HyPure^TM^ molecular biology grade water, 2.5 μL 10X reaction buffer (200 mM Tris-HCl, 500 mM KCl, pH 8.4), 1 μL MgCl_2_ (50 mM), 05. μL dNTPs mix (10 mM), 0.5 μL of both forward (10 mM) and reserve (10 mM) equimolar mixes, 0.5 μL Invitrogen’s Platinum Taq polymerase (5 U) and 2 μL of DNA. Final reaction volume totaled 25 μL.

PCR protocol largely followed Rivera et al. [15] with minor adjustments. Instead of thirty cycles of denaturation at 95°C for 45 seconds, annealing at 55°C for 45 seconds and extension at 72°C for 45 seconds [15], this study increased the number of cycles to thirty-five. PCR amplification was also performed in two-steps, with the second PCR using 2 μL of amplicons from the first PCR instead of DNA, and Illumina-tailed primers. All PCRs were completed in Eppendorf Mastercycler ep gradient S thermal cycler. Successful amplification was confirmed using 1.5% agarose gel electrophoresis before purifying second PCR amplicons with the MinElute Purification kit (Qiagen). The next step was quantifying purified samples with a QuantIT PicoGreen daDNA assay kit and using these values to normalize all samples to 3 ng/μL. Samples were then indexed and pooled before purifying with AMpure magnetic beads. QuantIT PicoGreen daDNA assay kit was once again used to quantify the library and Bioanalyzer was used to determine fragment length. The library was diluted to 4 nM and 10% PhiX was added before being sequenced using Illumina MiSeq with a V3 MiSeq sequencing kit (300 X 2; MS-102-2003).

### Bioinformatic processing

Illumina MiSeq paired-end reads were processed using the SCVURL rbcL metabarcode pipeline-1.0.2 pipeline available from https://github.com/terrimporter/SCVURL_rbcL_metabarcode_pipeline. SCVURL is an automated snakemake [47] bioinformatic pipeline that runs in a conda [48] environment. SeqPrep v1.3.2 [49] was used to pair raw reads requiring a minimum Phred score of 20 to ensure 99% base-calling accuracy. CUTADAPT v2.6 was used to trim primers from sequences, leaving a minimum fragment length of at least 150 base pairs [50]. Global exact sequence variant (ESV) [51] analysis was performed on the primer-trimmed reads. Reads were dereplicated using the ‘derep_fulllength’ command with the ‘sizein’ and ‘sizeout’ options of VSEARCH v2.14.1 [52]. VSEARCH was also used to denoise the data using the unoise3 algorithm [53]. These steps were taken to remove sequences with errors, chimeric sequences, and rare reads (singletons or doubletons) [54]. ESVs were classified using the rbcL diatom Classifier available from https://github.com/terrimporter/rbcLdiatomClassifier. Reference rbcL sequences were downloaded from the Diat.barcode project [55, 56] and reformatted to train the naive Bayesian classifier to make rapid, accurate taxonomic assignments [57]. This method makes assignments to the species rank and produces a statistical measure of confidence for each taxon up to the domain rank to help reduce false positive taxonomic assignments. We used a 0.40 bootstrap support cutoff at the genus rank and 0.90 bootstrap support cutoff at the species rank. We expect these taxonomic assignments to be correct 90% of the time assuming that target taxa are present in the reference database.

### Statistical analysis

RStudio was used to analyze the data [58]. To account for variable reads within the library each sample was normalized to the 15^th^ percentile using the ‘rrarefy’ function in the vegan package [59, 60].

ESV richness across the various sampling and status categories was calculated to assess differences between the methods and sites. A non-metric multi-dimensional (NMDS) analysis on Sorensen dissimilarities (binary Bray-Curtis) was conducted using the vegan ‘metaMDS’ function to determine if sampling method or site status created variation in community structure [5]. A scree plot was run using the ‘dimcheckMDS’ command from the goeveg package to determine the number of dimensions (k = 2) to use with vegan metaMDS function [61]. Shephard’s curve and goodness of fit calculations were calculated using the vegan ‘stressplot’ and ‘goodness’ functions. The vegan ‘vegdist’ command was used to build a Sorensen dissimilarity matrix. We checked for heterogeneous distribution of dissimilarities using the ‘betadisper’ function. We used the ‘adonis’ function to perform a permutational analysis of variance (PERMANOVA). PERMANOVA was performed on conventional sampling methods (periphyton collection) and kick-net methods, as well as site status to test for significant interactions between groups [62].

To maintain a balanced design during statistical testing, we pooled all periphyton sampling into one sample type (conventional) and maintained kick-net samples as a separate sample type, then tested for interactions between collection method and site status within sites. Nestedness and turnover of between kick-net and conventional samples were calculated using R package betapart function ‘beta-multi’ [63]. We used the method described by Baselga and Orme [63] that uses the ‘beta.sample’ function to account for unequal sample sizes and to visualize the nestedness and turnover components of beta diversity (Jaccard dissimilarity) across sites, methods, and site status. The number of diatom family ESVs detected from kick-net or pooled conventional samples was also plotted. A dendrogram of diatom families detected was plotted using RAWGraphs (app.rawgraphs.io) and color-coded to show the samples the families were detected in [64]. Lastly, the number of reads detected from diatom species were visualized using a heatmap generated using geom_tile (ggplot) in R.

### Taxonomic validation

Once diatoms had been classified to the species level, we searched for each unique species on the Diatoms of North America Database (NADED; https://diatoms.org/), to confirm the species were associated with freshwater and to collect additional ecological information. For each query, we collected NADED identification code, habitat preference (benthic/planktonic), and Biological Condition Gradient (BCG) score (1–5). Appreciating that the BCG model was developed for California (US), we also referred to the Eastern Canadian Diatom Index (IDEC: Indice Diatomées de l'Est du Canada [65]) to collect information on diatom classes.

## Results

After bioinformatic processing, we generated 4,284 ESVs (2,166,203 reads). After taxonomic filtering (keeping ESVs assigned to phylum Bacillariophyta only), a total of 4,197 diatom ESVs (2,153,099 reads) were retained for data analysis. Read coverage per sample after normalisation (15^th^ percentile cut-off) was 38,325.

Since the rarefaction curves plateau, this indicated that the sequencing depth was sufficient to capture the ESV diversity in our PCRs (S2 Fig in S1 File). In terms of the top 10 orders identified, the order Naviculales represented 35.1% of ESVs (34% of reads) and Bacillariales represented 19.2% of ESVs (15.6% of reads; S3 Fig in S1 File).

### Taxonomic coverage

**Fig 2:**
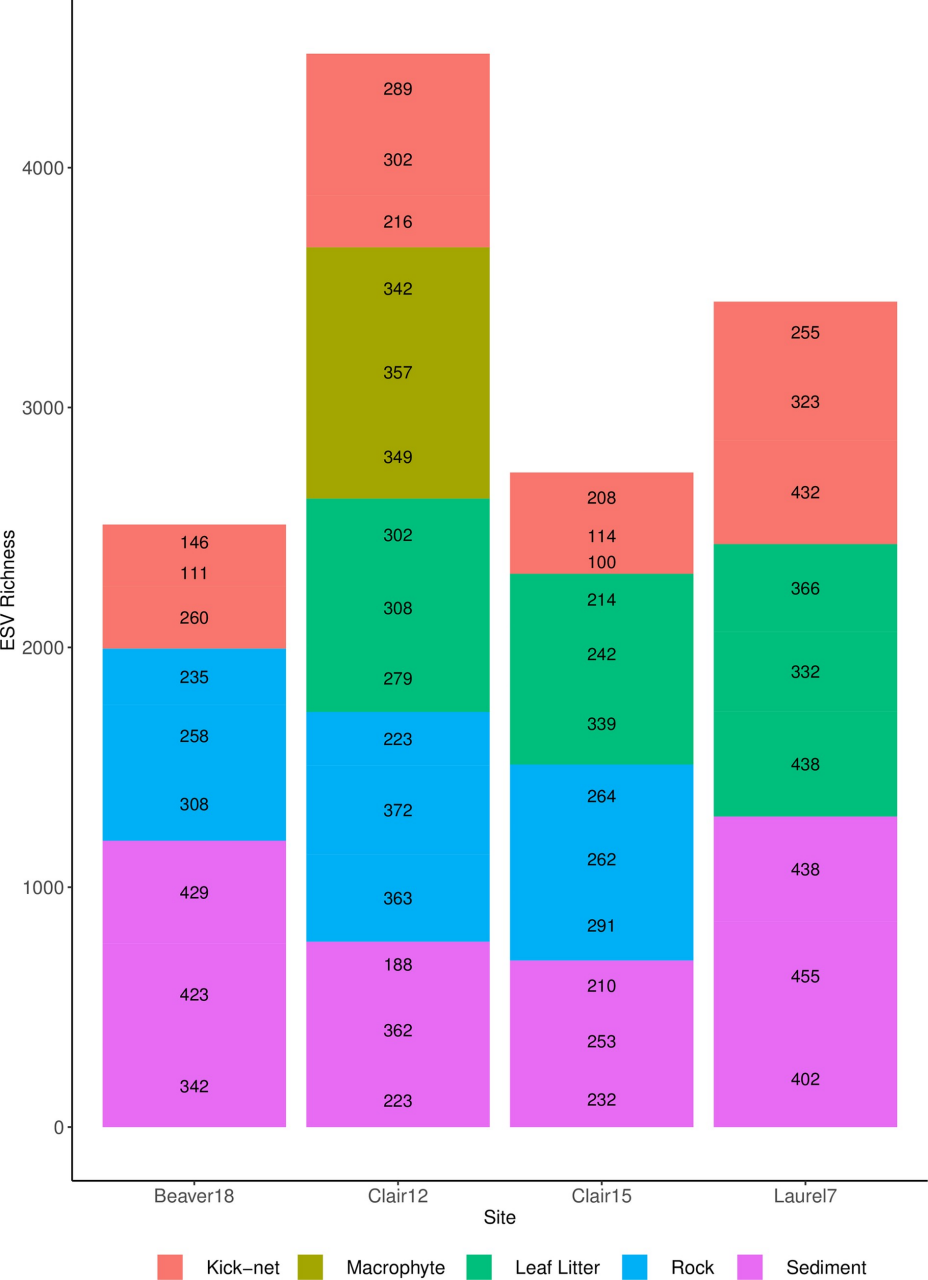
ESV richness varies across different sample types. Methods refer to the different sampling approaches analyzed (i.e. Kick-net, Macrophyte, Leaf Litter, Rock and Sediment). ESV scores are listed in replicate order (1–3), top to bottom of each microhabitat bar. Based on rarefied data.

In terms of taxonomic assignment, we identified a total of 1 phyla (Bacillariophyta), 8 classes, 32 orders,44 families and 58 genera at the 95% correct assignment level. At the 90% correct assignment level, we identified 165 unique diatom species. ESV richness varied across different sampling methods (Fig 2). Mean overall ESV richness was used to calculate alpha diversity which displayed very similar values for all sampling methods across the four sites (S4 Table in S1 File). Averaged across sites, kick-net samples produced the lowest mean ESV richness (230 ± 90), with sediment samples producing the highest average ESV richness (332 ± 97).

**Fig 3:**
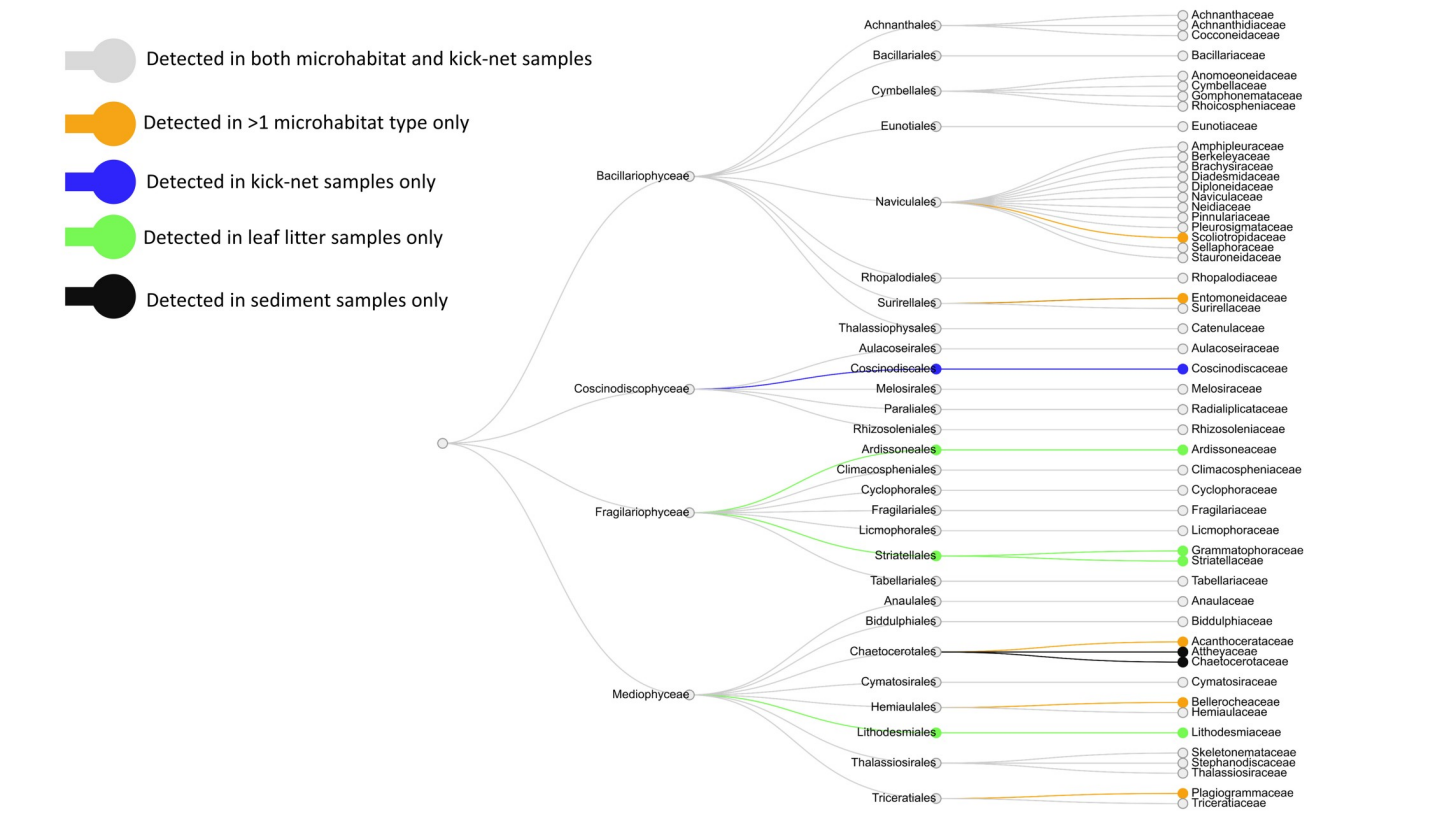
A majority of diatom families were detected in both microhabitat and kick-net samples. Left to right: Phylum (Bacillariophyta), Class, Order, Family.

Through investigating diatom orders and families, a majority of families detected were present in all microhabitats and kick-net samples (Fig 3). Three orders (‎Ardissoneales, Striatellales and Lithodesmiales) were solely present in leaf litter samples, two families (Attheyaceae and Chaetocerotaceae) were present only in sediment samples (Fig 3) and one order was solely present in kick-net samples (Coscinodiscales).

**Fig 4:**
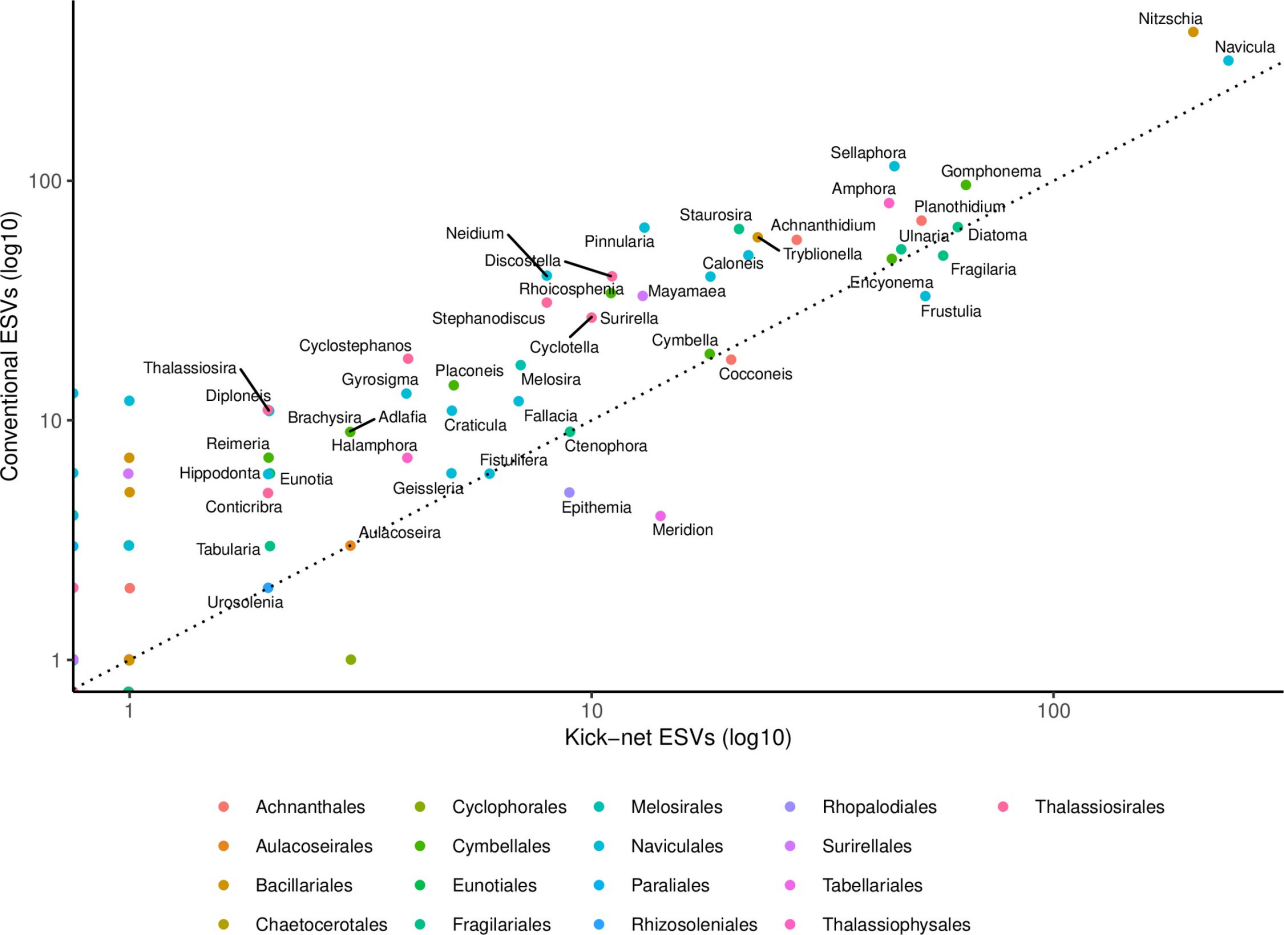
Number of ESVs detected from genera detected from kick-net versus conventionally sampled diatoms are similar. The points are color-coded for the orders detected in this study. A 1:1 correspondence line (dotted) is also shown. A log10 scale is shown on each axis to improve the spread of points with small values. Based on rarefied data.

In terms of diatom genera, some of the confidently identified genera represented by 2 or more sequence variants, identified from kick-net and conventional samples, included: *Nitzschia* (Bacillariales), *Navicula* (Naviculales), *Amphora* (Thalassiophysales) and *Ulnaria* (Licmophorales; Fig 4).

### Diatom diversity by method and site status

**Fig 5:**
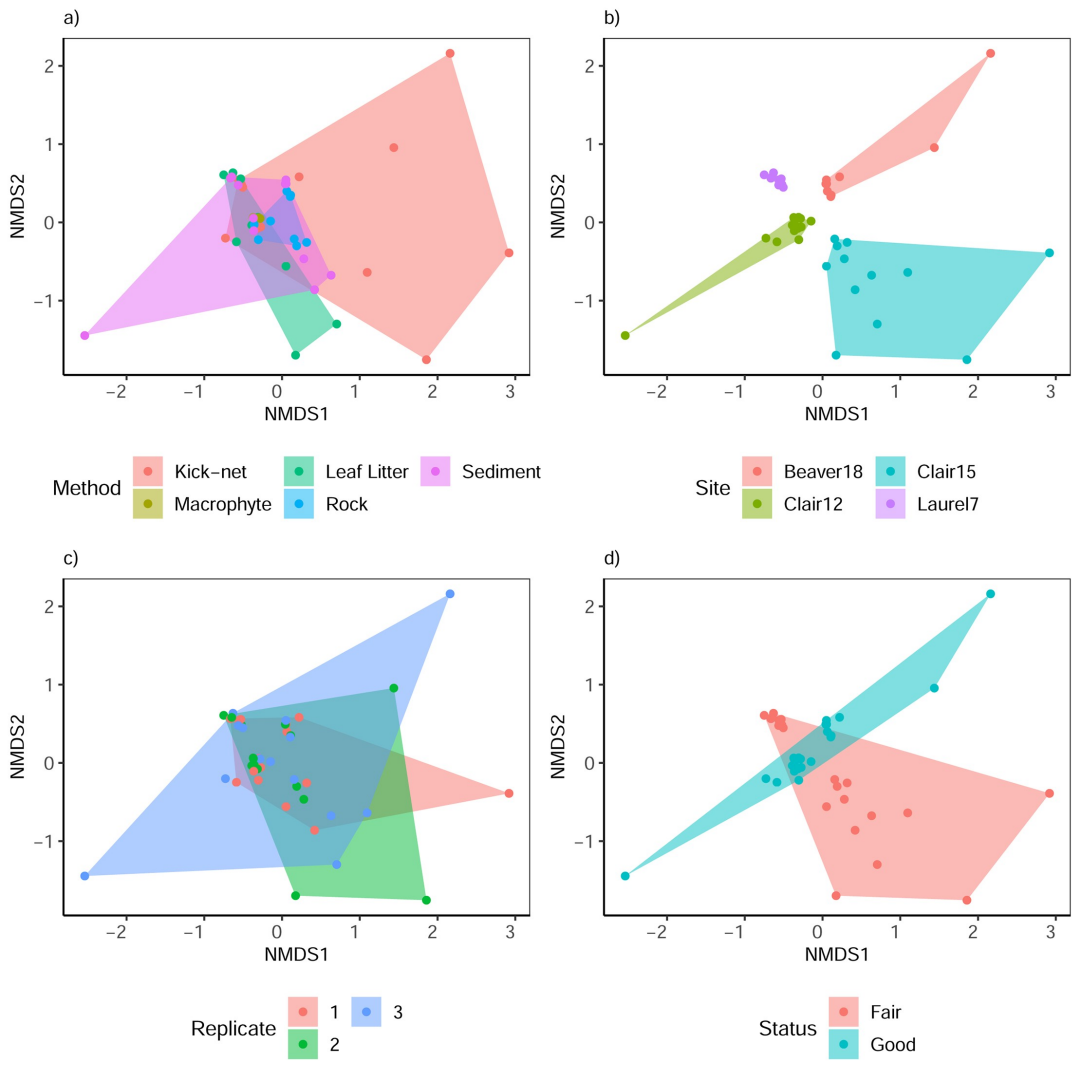
Non-metric multi-dimensional scaling plots show clustering mainly due to site and status. Specifically, a) binary Bray Curtis (Sorensen) dissimilarities overlapping across different sampling approaches, b) clustering by site, c) overlap between replicates, and d) clustering based on habitat quality status (stress = 0.109, R^2^ = 0.98). Based on rarefied data.

NMDS plots showed that replicates clustered close together for site and status, with overlap observed between sampling methods and replicates (Fig 5). When pooling conventional periphyton samples (i.e. macrophyte, leaf litter, rock, and sediment) at each site, there remained overlap between kick-net and conventional samples and samples also remained clustered by site and status (S4 Fig in S1 File). PERMANOVA of the pooled samples, shows that analyzing data from kick-net or conventional samples (method) explains 14% of the variation in Bray Curtis dissimilarities (p-value = 0.125), sampling site (site) explains 57% of the variation (p-value = 0.042) and habitat quality status (status) explains 15% of the variation observed (p-value = 0.333; S5 Table in S1 File). Across all groups, the nestedness component of beta diversity tended to be very low and overall beta diversity was driven by the very high turnover component. Diatom diversity across site status was similar, with slightly higher dissimilarity in ‘fair’ sites. Diatom diversity showed similar patterns across collection methods with slightly lower diversity recovered from rock scrapings. Diatom diversity did show site specific patterns with the lowest dissimilarity recovered from Laurel7 (fair) and the highest dissimilarity from Clair 15 (good) (S6 Fig in S1 File). Similarly, for beta diversities of communities aggregated by the "kick-net" and "conventional" collection method, there was also high turnover (0.91) and low nestedness (0.02) components with respect to overall Jaccard dissimilarity (0.93) across methods (S5 Fig in S1 File).

**Fig 6:**
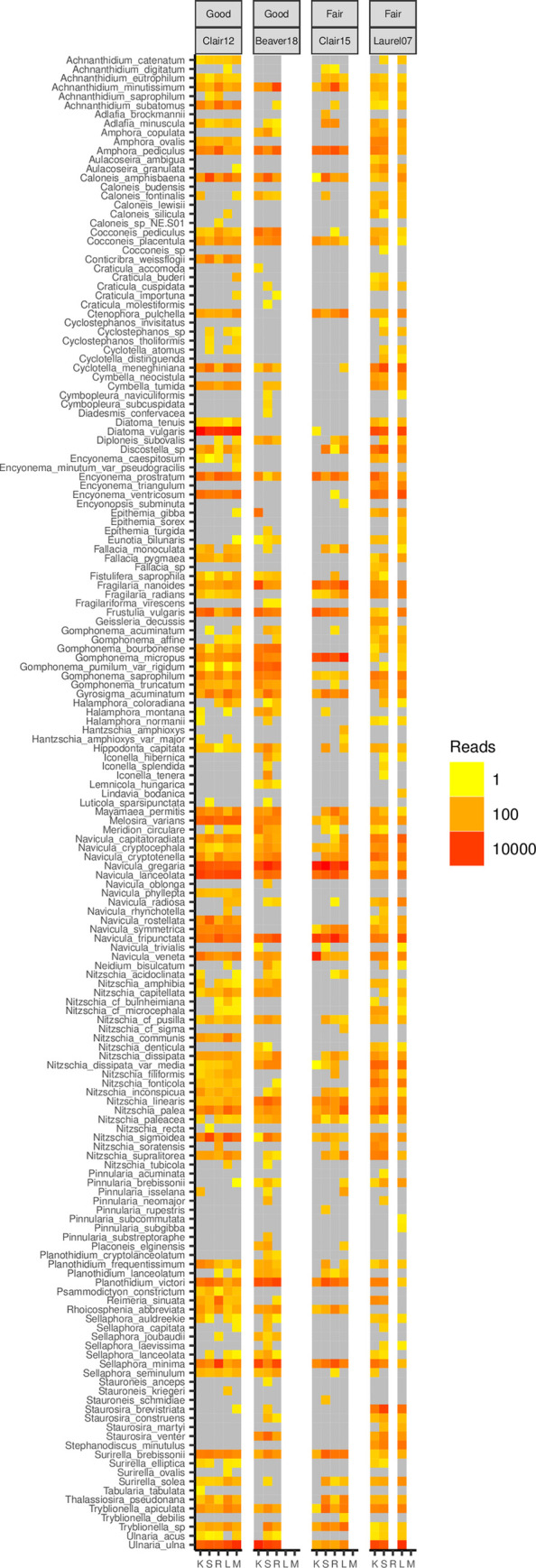
Samples detect similar diatom species across sampling methods and site status. Only ESVs taxonomically assigned to species with high confidence (bootstrap support > = 0.90 for 90% accuracy) are included. Plots are faceted by site + site status. Sampling methods: K = kick-net; R = rock scraping; L = leaf litter; M = macrophyte; S = sediment. White lanes indicate the corresponding microhabitat was not present at the site; grey lanes indicate species was not present within the particular site. For each site, three replicates for each sampling method are pooled. Based on normalized data.

For individual sample types (i.e. kick-net, macrophyte, leaf litter, rock, and sediment), the heatmap shows that kick-net samples are largely representative of the diversity of species detected within each conventional periphyton sampling method (Fig 6). In some cases, kick-net samples failed to detect diatom species which were present in conventional periphyton samples (e.g. *Gomphonema acuminatum* in Clair 12) and conversely, kick-net samples also detected species which were not detected in conventional periphyton samples (e.g. *Pinnularia isselana* in Clair12 and *Sellaphora seminulum* in Laurel 7; Fig 6). Similar assemblages of diatom communities were detected across both fair and good quality sites, and some species of diatoms were only detected in one site (e.g. *Stauroneis kriegeri* in Clair 12, *Lemnicola hungarica* in Beaver 18 and *Stauroneis schmidiae* in Clair 15; Fig 6).

**Fig 7:**
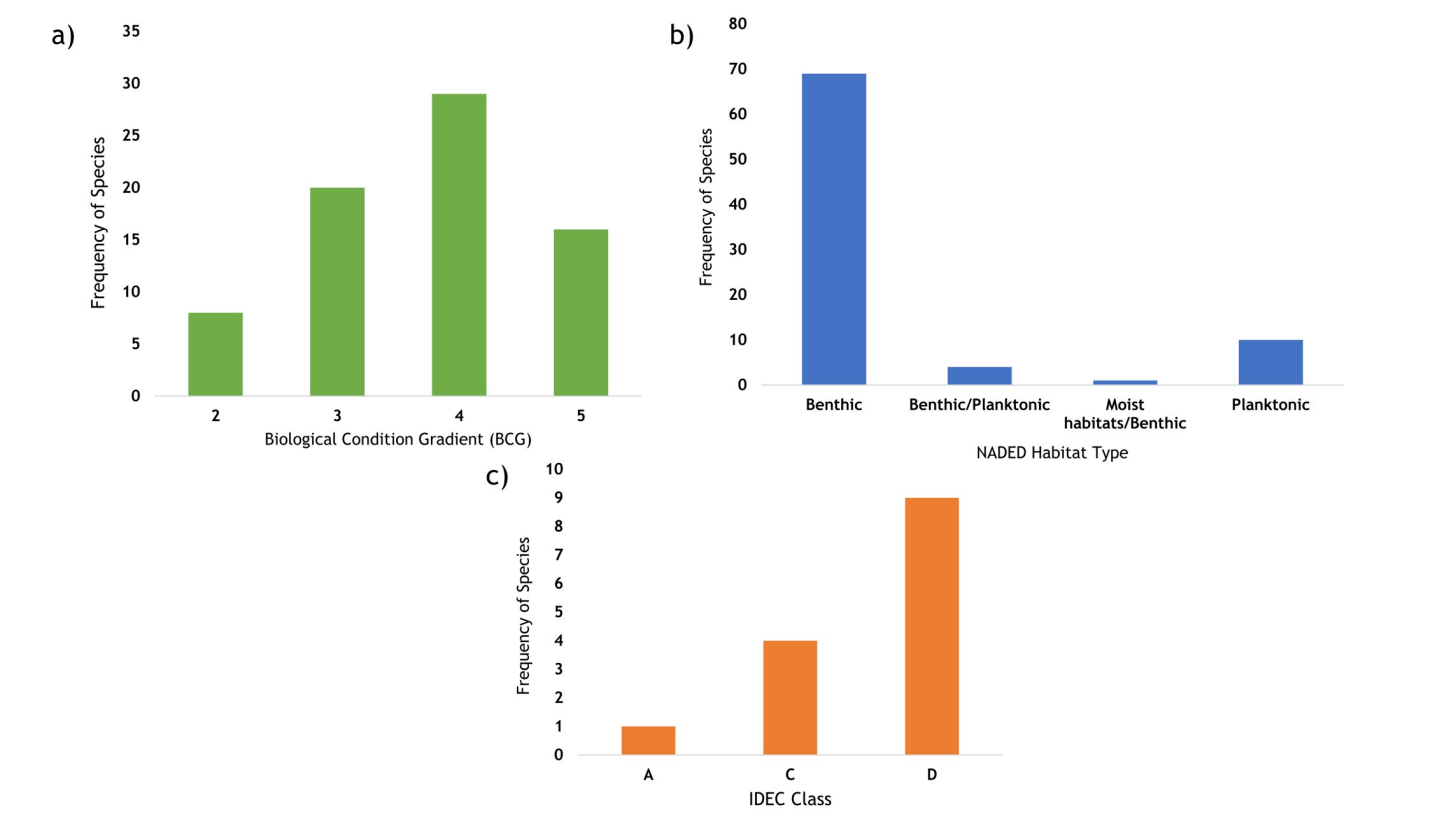
Frequency plots for diatom species identified in this study and present on the North American Diatom Database. a) Biological Condition Gradient (BCG) scores (number of diatom species = 73, 1 = specialist species, 2 = highly sensitive species, 3 = sensitive species, 4 = indiscriminate species and 5 = tolerant species); b) Habitat types (number of diatom species = 84); c) Eastern Canadian Diatom Index classes (number of diatom species = 14, A = reference condition, B = slightly altered, C = altered, D = severely altered conditions).

Out of the 165 unique diatom species identified, 88 were located on the Diatoms of North America Ecological Database (NADED; S6 Table in S1 File). Not all desired ecological information was available for each of these species, however all 88 were identified as being associated with freshwater. In terms of associated microhabitat, we detected 10 exclusively planktonic species, 8 of which were not detected using the rock scraping methodology. For BCG (1 = specialist species, 2 = highly sensitive species, 3 = sensitive species, 4 = indiscriminate species and 5 = tolerant species[66]), the majority of diatom species detected were classified as indiscriminate (Fig 7) however 28 species were classified as being highly sensitive/sensitive species. Of the highly sensitive species, three are nitrogen-fixing, and are typically found in nitrogen-poor environments.

## Discussion

The demand for high-quality, reproducible ecological data is increasing in conjunction with the degradation of ecosystems globally [67]. There is a need to further streamline existing biomonitoring methodologies without sacrificing the quality of data produced [4, 7, 40, 62]. With diatom assemblages providing a unique insight into the water quality status of lentic and lotic systems, fast-tracking diatom data collection for ecological assessments is a priority [15]. We have demonstrated that kick-net methodology with DNA metabarcoding provides sufficient taxonomic coverage to potentially be utilised to opportunistically assess diatom biodiversity in freshwater systems along with macroinvertebrates.

Kick-net sampling technique, whereby a zig-zag path is taken across the reach, provided sufficient representation of existing diatom community assemblages within site-specific microhabitats. Samples derived from the kick-net technique were highly comparable with conventional samples in terms of diatom taxa detected, despite the kick-net approach being more passive compared to direct periphyton collection. Specific diatom taxa are known to have ecological preferences for different freshwater microhabitats [68, 69]. For watershed-level health estimates, it is beneficial to be able to efficiently detect the diversity of diatom taxa present without directly sampling each microhabitat within a reach. We have demonstrated that kick-net methodology can sufficiently capture the existing diatom biodiversity, ground truthed by comparing assemblages detected with periphyton collection.

For conventional periphyton collection, taxonomic ID of diatoms often strictly focus on sub-species or species-level classifications, irrespective of sample processing cost and time [35]. However, the taxonomic identification process often lacks a validation process or an assignment score. The classifications of diatoms to species level can vary between taxonomists, depending on skill level and availability of taxonomic keys and their updates [70–72]. It has been noted that quantifying diatom classification error based on the analyst’s performance is difficult, as many factors can influence the results [73]. Inconsistencies between classified diatom datasets can misinform diatom taxon-specific water quality assessments [70] and there has been limited research to date concerning the ‘certainty’ and ‘precision’ of ecological classifications based on diatoms as bioindicators [74]. For DNA metabarcoding approaches, despite incomplete reference libraries being a limiting factor [75], it is possible to ascertain a quantifiable level of identification certainty not currently possible, or at least not widely reported, with conventional identification. Assignment methods such as the classifier approach we used in this study can provide statistical confidence of each assigned taxon (e.g. 90% bootstrap for species-level) and other approaches such as phylogenetic placement methods can provide phylogenetic relatedness of query sequences, which can aid identification especially when exact species are not available in reference sequence libraries [76].

Ultimately, the detection of bioindicator species is a key variable to consider when comparing biomonitoring methods, as these taxa are pivotal for detecting subtle differences in freshwater health [3, 5, 14]. *Navicula* contains some diatom species sensitive to herbicide exposure, which is a genus we observed in all sites and with all collection methods [77]. At the species level, highly sensitive/sensitive taxa were detected mostly with all collection methods, however for some rarer species such as *Epithemia gibba*, rock scraping method failed to detect this species despite presence in all other collection methods. Rock scrapings are commonly used as the sole collection method for diatoms [14, 15, 78, 79], which suggests that the kick-net approach facilitates the detection of ecological indicator taxa which otherwise may be missed from conventional sampling.

With respect to conventional approach of rock scraping, this approach is mostly biased towards benthic diatom species [26]. Depending on the research question, targeting only this group of taxa may suffice, however for sampling diatoms to answer questions regarding fine-scale freshwater health, it is essential to consider the presence of planktonic diatom species [80–83]. In terms of sampling method, benthic kick-net sampling detected more planktonic diatom species than rock scraping alone. A portion of the planktonic diatom species detected in this study were either sensitive or highly tolerant, highlighting the importance of planktonic diatom species presence in determining the extent of anthropogenic influence on a river system [82]. Warming-induced changes to freshwater ecosystems are known to favour the increase in abundance of planktonic diatom species in the genus *Cyclotella* over planktonic *Aulacoseira* and/or benthic *Fragilaria* species [84]. Ultimately, with the current trajectory of climate-induced warming, this is likely to result in long-term ecological changes through shifts in diatom community assemblages [82, 84]. To date, the monitoring of planktonic diatoms is conducted through sediment traps/water column sampling [84], however here we have demonstrated the ability of benthic kick-net sampling to detect planktonic diatoms as well.

## Conclusion

Overall, this study found that benthic kick-net methodology enables a robust and detailed assessment of freshwater diatom communities. This methodology is a scalable option for generating a holistic insight into the health of freshwater systems. The high similarity of diatom taxa detected between methods and ecological inference from the species-level classifications of diatoms, demonstrates that this rapid method can provide accurate, fine-resolution taxonomic results. Future research should examine the duo-analyses approach of macroinvertebrate and diatom communities from a single kick-net sample, to determine reproducibility of multi-taxa targeting with this method. Additionally, future studies should consider exploring the use of multiple markers (i.e. rbcL cpDNA versus 18S rRNA gene), to address level of taxonomic resolution that can be obtained with these markers commonly used for diatom DNA barcoding.

## Supporting information

S1 File (DOCX)
