# Supplementary material for: Freshwater diatom biomonitoring through benthic kick-net metabarcoding
Source: PLoS One. 2020 Nov 18;15(11):e0242143. doi: 10.1371/journal.pone.0242143 (PMC7673570; doi:10.1371/journal.pone.0242143)
Supplement: S1 File — (DOCX) [file pone.0242143.s001.docx]

**Freshwater diatom biomonitoring through benthic kick-net metabarcoding**

Victoria **Carley** Maitland, Chloe V. Robinson, Teresita M. Porter, Mehrdad Hajibabaei*.

* Corresponding author: [mhajibab@uoguelph.ca](mailto:mhajibab@uoguelph.ca)

**S1 Table. Information on study sites, including GPS coordinates and site status.**

|  | Quality Status | Latitude | Longitude | Number of kick-net samples | Microhabitats Sampled |
| --- | --- | --- | --- | --- | --- |
| Beaver 18 | Good | 43.4920012 | -80.609783 | 3 | 2 (Rock and Sediment) |
| Clair 12 | Fair | 43.4654088 | -80.571321 | 3 | 4 (Rock, Leaf Litter, Macrophyte and Sediment) |
| Clair 15 | Good | 43.46290086 | -80.58467794 | 3 | 3 (Rock, Leaf Litter and Sediment) |
| Laurel 7 | Fair | 43.4707269 | -80.556274 | 3 | 2 (Leaf Litter and Sediment) |

**S2 Table. Outline of collections methods used in this study.** Samples for periphyton scraping were taken from a depth no greater than 1m [23].

| Sampling Type | Collection Method Description |
| --- | --- |
| Kick-net | 400 µm mesh net and frame attached to a pole was placed in water with the mouth facing upstream. Sampler kicked their feet to disturb benthos while moving upstream in a zig-zag pattern for a total of three minutes. If obstruction occurred, a timer was stopped until net was once again free, and kicking could recommence. When time elapsed, the net was lifted from the water and drained before placing contents into 1 L sample jar. |
| Sediment | The top 2 mm of sediment was suctioned using a 50 mL pipette until 10 mL of sample was collected. Contents were then dispensed into a 1 L sample jar and sampler then repeated these steps four more times before closing and storing sample jar. |
| Rocks | Sampler randomly selected 5 rocks from the reach, all approximately 10-20 cm in intermediate axis. Rocks were placed in a tub and transported to the stream bank where a minimum of 100 cm^2^ [27] of biofilm was scraped into the sampling jar using a previously sterilised toothbrush. 100% ethanol in a squirt bottle was also used to direct the scrapings into the 1 L sampling jar. |
| Macrophytes/Leaf Litter | Macrophytes and leaf litter samples were collected in an identical manner. Small handfuls of macrophytes representative of the reach and leaf litter was collected five times and placed into a 1 L sampling jar ¼ filled with water. Once all foliage was placed in the jar, lid was closed, and sample was shaken vigorously for 45 seconds. After time was complete, macrophytes and/or leaf litter was gently rubbed to remove any remaining periphyton before being discarded. Sample jar containing periphyton-water mixture was then closed and set aside. |

**S3 Table. Summary table of decontamination and sterilisation procedures undertaken for the equipment in this study.**

| Equipment | Decontamination/Sterilisation Procedure |
| --- | --- |
| Kick-nets | Nets were submerged in 10% bleach solution for 20 minutes then soaked in water for 20 minutes. When complete, nets were placed in clean garbage bags until use. |
| Sample jars | Sample jars were cleaned with ELIMINase® (VWR, Canada) and scrubbed with a soft bristle brush before being rinsed with deionized water. Once clean, jars were treated with UV light for 30 minutes then closed and sealed in plastic bags until use. |
| Toothbrushes | Toothbrushes were removed from original packaging and soaked in 10% bleach solution for 20 minutes, then water for an additional 20 minutes. Toothbrushes were then treated with UV light for 30 minutes and sealed in plastic bags until use. |

**S4 Table. Mean ESV richness (Bacillariophyta only) for each sample type across the four sites.** Data was pooled across replicates. Based on normalised data.

|  | Beaver 18 | Clair 12 | Clair 15 | Laurel 7 | Average (± SD) |
| --- | --- | --- | --- | --- | --- |
| Kick-net | 172 | 269 | 141 | 337 | 230 (± 90) |
| Macrophyte | N/A | 349 | N/A | N/A | 349 (± 0) |
| Leaf litter | N/A | 296 | 265 | 379 | 313 (± 59) |
| Rock | 267 | 319 | 272 | N/A | 286 (± 29) |
| Sediment | 398 | 268 | 232 | 431 | 332 (± 97) |

N/A: sample not collected; SD: standard deviation.

**S5 Table. rbcL exact sequence variants (ESVs) are not significantly different between sampling methods (kick-net versus conventional periphyton sampling).** No significant beta dispersion was detected within groups (method, site, status). The only significant difference detected was rbcL ESVs between sites and status. Summary of PERMANOVA results based on a Sorensen dissimilarity matrix of rbcL ESVs. Significant p-values are bolded.

| Source of variation | Df | MS | F | R^2^ | P |
| --- | --- | --- | --- | --- | --- |
| A) sor ~ method (strata = site) | | | | | |
| Preservative | 1 | 0.285 | 0.955 | 0.137 | 0.125 |
| Residuals | 6 | 0.298 |  | 0.862 |  |
| Total | 7 |  |  | 1.000 |  |
|  |  |  |  |  |  |
| B) sor ~ site (strata = method) | | | | | |
| Site | 3 | 0.394 | 1.770 | 0.570 | **0.042*** |
| Residuals | 4 | 0.222 |  | 0.430 |  |
| Total | 7 |  |  | 1.000 |  |
|  |  |  |  |  |  |
| C) sor ~ status (strata = method) | | | | | |
| Status | 1 | 0.314 | 1.069 | 0.151 | 0.333 |
| Residuals | 6 | 0.294 |  | 0.848 |  |
| Total | 7 |  |  | 1.000 |  |
|  |  |  |  |  |  |
| D) sor ~ status*method (strata = site) | | | | | |
| Status | 1 | 0.314 | 0.971 | 0.151 | 0.250 |
| Method |  | 0.285 | 0.881 | 0.137 | 0.125 |
| Status:Method |  | 0.183 | 0.568 | 0.088 | 1.000 |
| Residuals | 6 | 0.323 |  | 0.623 |  |
| Total | 7 |  |  | 1.000 |  |

sor (binary Bray Curtis sample by ESV matrix); Method (kick-net or conventional); Site (Clair 12, Clair 15, Beaver 18 or Laurel 07); Status (Fair or Good)

**S6 Table. Diatom species information for species detected in this study, obtained from Diatoms of North America database and Eastern Canadian Diatom Index (IDEC: Indice Diatomées de l'Est du Canada; Neutral).**

| Species | NADED ID* | Habitat | [BCG](https://diatoms.org/practitioners/how-and-where-do-diatoms-live-autecology-tags)** | IDEC Class*** | Detection+ | NADED Citation |
| --- | --- | --- | --- | --- | --- | --- |
| *Achnanthidium eutrophilum* | 1046 | Benthic | N/A | N/A | All | Potapova, M., Spaulding, S., Edlund, M. (2008). Achnanthidium. In Diatoms of North America. Retrieved July 24, 2020, from https://diatoms.org/genera/achnanthidium |
| *Achnanthidium minutissimum* | 1010 | Benthic | 3 | A | All | Potapova, M. (2009). Achnanthidium minutissimum. In Diatoms of North America. Retrieved July 24, 2020, from https://diatoms.org/species/achnanthidium_minutissimum |
| *Amphora copulata* | 7075 | Benthic | 4 | N/A | K, S, L, R | Stepanek, J., Kociolek, P. (2011). Amphora copulata. In Diatoms of North America. Retrieved July 24, 2020, from https://diatoms.org/species/amphora_copulata |
| *Amphora ovalis* | 7001 | Benthic | 4 | N/A | All | Stepanek, J., Kociolek, P. (2011). Amphora ovalis. In Diatoms of North America. Retrieved July 24, 2020, from https://diatoms.org/species/amphora_ovalis |
| *Amphora pediculus* | 7043 | Benthic | 4 | N/A | All | Stepanek, J., Kociolek, P. (2011). Amphora pediculus. In Diatoms of North America. Retrieved July 24, 2020, from https://diatoms.org/species/amphora_pediculus |
| *Aulacoseira ambigua* | 10008 | Planktonic | 3 | N/A | K, S | Potapova, M., English, J. (2010). Aulacoseira ambigua . In Diatoms of North America. Retrieved July 24, 2020, from https://diatoms.org/species/aulacoseira_ambigua |
| *Aulacoseira granulata* | 10018 | Planktonic | 5 | N/A | K, S, L M | Potapova, M., English, J. (2010). Aulacoseira granulata. In Diatoms of North America. Retrieved July 24, 2020, from https://diatoms.org/species/aulacoseira_granulata |
| *Caloneis amphisbaena* | 12023 | Benthic | 4 | N/A | All | Haueter, J. (2014). Caloneis amphisbaena. In Diatoms of North America. Retrieved July 24, 2020, from https://diatoms.org/species/caloneis_amphisbaena |
| *Caloneis lewisii* | 12014 | Benthic |  | N/A | K, S, L | Keith, M., Rosen, B. (2012). Caloneis lewisii. In Diatoms of North America. Retrieved July 24, 2020, from https://diatoms.org/species/caloneis_lewisii |
| *Caloneis silicula* | 12010 | Benthic | 3 | N/A | K, S, L | Kociolek, P. (2011). Caloneis silicula. In Diatoms of North America. Retrieved July 24, 2020, from https://diatoms.org/species/caloneis_silicula |
| *Cocconeis pediculus* | 16011 | Benthic | 4 | C | All | Grubaugh, C., Potapova, M. (2012). Cocconeis pediculus . In Diatoms of North America. Retrieved July 24, 2020, from https://diatoms.org/species/cocconeis_pediculus |
| *Cocconeis placentula* | 16004 | Benthic | 4 | D | All | Potapova, M., Spaulding, S. (2013). Cocconeis placentula sensu lato. In Diatoms of North America. Retrieved July 24, 2020, from https://diatoms.org/species/cocconeis_placentula |
| *Conticribra weissflogii* | 70008 | Planktonic | 4 | N/A | All | Kociolek, P. (2011). Thalassiosira weissflogii. In Diatoms of North America. Retrieved July 24, 2020, from https://diatoms.org/species/thalassiosira_weissflogii |
| *Craticula accomoda* | 21003 | Benthic | 5 | N/A | K | Bishop, I., Burge, D. (2015). Craticula accomoda. In Diatoms of North America. Retrieved July 24, 2020, from https://diatoms.org/species/craticula_accomoda |
| *Craticula buderi* | N/A | Benthic | N/A | N/A | K, S, M | Bahls, L., Kociolek, P. (2012). Craticula buderi. In Diatoms of North America. Retrieved July 24, 2020, from https://diatoms.org/species/craticula_buderi |
| *Craticula molestiformis* | 21015 | N/A | 5 | N/A | S | Vaccarino, M., LaLiberte, G. (2015). Craticula molestiformis. In Diatoms of North America. Retrieved July 24, 2020, from https://diatoms.org/species/craticula_molestiformis |
| *Craticula cuspidata* | 21004 | Benthic | 4 | N/A | K, S, L | Fuelling, L., LaLiberte, G. (2011). Craticula cuspidata. In Diatoms of North America. Retrieved July 24, 2020, from https://diatoms.org/species/craticula_cuspidata |
| *Ctenophora pulchella* | 201001 | Benthic | 4 | N/A | All | Jones, J. (2013). Ctenophora pulchella. In Diatoms of North America. Retrieved July 24, 2020, from https://diatoms.org/species/ctenophora_pulchella |
| *Cyclostephanos invisitatus* | 19002 | Planktonic | 4 | N/A | S | Burge, D., Edlund, M. (2015). Cyclostephanos invisitatus. In Diatoms of North America. Retrieved July 24, 2020, from https://diatoms.org/species/cyclostephanos_invisitatus |
| *Cyclostephanos tholiformis* | 19001 | Planktonic | 3 | N/A | S, M | Burge, D., Edlund, M. (2015). Cyclostephanos tholiformis. In Diatoms of North America. Retrieved July 24, 2020, from https://diatoms.org/species/cyclostephanos_tholiformis |
| *Cyclotella atomus* | 20001 | Planktonic | 5 | N/A | S, L, M | Lowe, R. (2015). Cyclotella atomus. In Diatoms of North America. Retrieved July 24, 2020, from https://diatoms.org/species/cyclotella_atomus |
| *Cyclotella distinguenda* | 20063 | Planktonic | N/A | N/A | S, L | Lowe, R., Manoylov, K. (2011). Cyclotella distinguenda. In Diatoms of North America. Retrieved July 24, 2020, from https://diatoms.org/species/cyclotella_distinguenda |
| *Cyclotella meneghiniana* | 20007 | Planktonic | 5 | D | All | Lowe, R., Kheiri, S. (2015). Cyclotella meneghiniana. In Diatoms of North America. Retrieved July 24, 2020, from https://diatoms.org/species/cyclotella_meneghiniana |
| *Cymbella neocistula* | N/A | Benthic | N/A | N/A | K, S, L | White, C. (2011). Cymbella neocistula. In Diatoms of North America. Retrieved July 24, 2020, from https://diatoms.org/species/cymbella_neocistula |
| *Cymbopleura naviculiformis* | 190005 | Benthic | 3 | N/A | S, L | Bahls, L. (2012). Cymbopleura naviculiformis. In Diatoms of North America. Retrieved July 24, 2020, from https://diatoms.org/species/cymbopleura_naviculiformis |
| *Cymbopleura subcuspidata* | 190007 | Benthic | N/A | N/A | S | Bahls, L. (2012). Cymbopleura subcuspidata. In Diatoms of North America. Retrieved July 24, 2020, from https://diatoms.org/species/cymbopleura_subcuspidata |
| *Diadesmis confervacea* | 197001 | Benthic | 5 | N/A | All | Kociolek, P. (2011). Diadesmis confervacea. In Diatoms of North America. Retrieved July 24, 2020, from https://diatoms.org/species/diadesmis_confervacea |
| *Diatoma tenuis* | 27012 | Benthic/Planktonic | 2 | N/A | All | Potapova, M. (2010). Diatoma tenuis. In Diatoms of North America. Retrieved July 24, 2020, from https://diatoms.org/species/diatoma_tenuis |
| *Diatoma vulgaris* | 27013 | Benthic | 3 | N/A | All | Potapova, M. (2009). Diatoma vulgaris. In Diatoms of North America. Retrieved July 24, 2020, from https://diatoms.org/species/diatoma_vulgaris |
| *Encyonema minutum var pseudogracilis* | N/A | Benthic | N/A | N/A | M | Bishop, I. (2017). Encyonema minutum var. pseudogracilis. In Diatoms of North America. Retrieved July 24, 2020, from https://diatoms.org/species/encyonema_minutum_var._pseudogracilis |
| *Epithemia gibba* | 58001 | Benthic/rare | 2 | N/A | K, S, L, M | Kociolek, P. (2011). Epithemia gibba. In Diatoms of North America. Retrieved July 24, 2020, from https://diatoms.org/species/epithemia_gibba |
| *Epithemia sorex* | 32006 | Benthic | 2 | N/A | L | Lowe, R. (2010). Epithemia sorex. In Diatoms of North America. Retrieved July 24, 2020, from https://diatoms.org/species/epithemia_sorex |
| *Epithemia turgida* | 32004 | Benthic | 2 | N/A | S, L | Lowe, R. (2010). Epithemia turgida. In Diatoms of North America. Retrieved July 24, 2020, from https://diatoms.org/species/epithemia_turgida |
| *Eunotia bilunaris* | 33185 | Moist habitats/Benthic | 3 | N/A | All | Burge, D., Edlund, M. (2015). Eunotia bilunaris. In Diatoms of North America. Retrieved July 24, 2020, from https://diatoms.org/species/eunotia_bilunaris |
| *Fallacia monoculata* | N/A | Benthic | N/A | N/A | All | Bishop, I. (2016). Pseudofallacia monoculata. In Diatoms of North America. Retrieved July 24, 2020, from https://diatoms.org/species/fallacia_monoculata |
| *Fallacia pygmaea* | 115001 | Benthic | 5 | N/A | All | Kociolek, P. (2011). Fallacia pygmaea. In Diatoms of North America. Retrieved July 24, 2020, from https://diatoms.org/species/fallacia_pygmaea |
| *Fragilariforma virescens* | 192008 | Benthic | 2 | N/A | S, R | Morales, E., Spaulding, S. (2011). Fragilariforma virescens. In Diatoms of North America. Retrieved July 24, 2020, from https://diatoms.org/species/fragilariforma_virescens |
| *Frustulia vulgaris* | 35011 | Benthic | 4 | N/A | All | Kociolek, P., Graeff, C. (2011). Frustulia vulgaris. In Diatoms of North America. Retrieved July 24, 2020, from https://diatoms.org/species/frustulia_vulgaris |
| *Geissleria decussis* | 210003 | Benthic | 4 | N/A | K, S | Potapova, M. (2009). Geissleria decussis. In Diatoms of North America. Retrieved July 24, 2020, from https://diatoms.org/species/geissleria_decussis |
| *Gomphonema acuminatum* | 37001 | Benthic | 3 | N/A | All | Kociolek, P. (2011). Gomphonema acuminatum. In Diatoms of North America. Retrieved July 24, 2020, from https://diatoms.org/species/gomphonema_acuminatum |
| *Gomphonema truncatum* | 37022 | Benthic | 3 | N/A | All | Kociolek, P. (2011). Gomphonema truncatum. In Diatoms of North America. Retrieved July 24, 2020, from https://diatoms.org/species/gomphonema_truncatum |
| *Gyrosigma acuminatum* | 38001 | Benthic | 4 | N/A | All | Chaput, M. (2014). Gyrosigma acuminatum. In Diatoms of North America. Retrieved July 24, 2020, from https://diatoms.org/species/gyrosigma_acuminatum |
| *Halamphora montana* | 7042 | Benthic | 4 | N/A | K, S, R | Stepanek, J., Kociolek, P. (2011). Halamphora montana. In Diatoms of North America. Retrieved July 24, 2020, from https://diatoms.org/species/halamphora_montana |
| *Halamphora normanii* | 7007 | Benthic | 4 | N/A | K, S, L | Stepanek, J. (2011). Halamphora normanii. In Diatoms of North America. Retrieved July 24, 2020, from https://diatoms.org/species/halamphora_normanii |
| *Hippodonta capitata* | 213001 | Benthic | 4 | D | All | Potapova, M. (2011). Hippodonta capitata. In Diatoms of North America. Retrieved July 24, 2020, from https://diatoms.org/species/hippodonta_capitata |
| *Lemnicola hungarica* | 188001 | Benthic | 4 | N/A | K, S, R | Potapova, M. (2010). Lemnicola hungarica. In Diatoms of North America. Retrieved July 24, 2020, from https://diatoms.org/species/lemnicola_hungarica |
| *Lindavia bodanica* | 208004 | Planktonic | N/A | N/A | L | Burge, D., Edlund, M. (2017). Lindavia bodanica. In Diatoms of North America. Retrieved July 24, 2020, from https://diatoms.org/species/lindavia_bodanica |
| *Melosira varians* | 44073 | Benthic/Planktonic | 4 | D | All | Potapova, M. (2009). Melosira varians. In Diatoms of North America. Retrieved July 24, 2020, from https://diatoms.org/species/melosira_varians |
| *Meridion circulare* | 45001 | Benthic | 2 | N/A | All | Kociolek, P. (2011). Meridion circulare. In Diatoms of North America. Retrieved July 24, 2020, from https://diatoms.org/species/meridion_circulare |
| *Navicula capitatoradiata* | 46661 | Benthic | 3 | C | All | Rushforth, S., Spaulding, S. (2010). Navicula capitatoradiata. In Diatoms of North America. Retrieved July 24, 2020, from https://diatoms.org/species/navicula_capitatoradiata |
| *Navicula cryptocephala* | 46014 | Benthic | 4 | C | All | Potapova, M. (2011). Navicula cryptocephala. In Diatoms of North America. Retrieved July 24, 2020, from https://diatoms.org/species/navicula_cryptocephala |
| *Navicula gregaria* | 46023 | Benthic | 5 | D | All | Potapova, M. (2011). Navicula gregaria. In Diatoms of North America. Retrieved July 24, 2020, from https://diatoms.org/species/navicula_gregaria |
| *Navicula lanceolata* | 46859 | Benthic | 3 | N/A | All | Potapova, M. (2009). Navicula lanceolata. In Diatoms of North America. Retrieved July 24, 2020, from https://diatoms.org/species/navicula_lanceolata |
| *Navicula oblonga* | 46832 | Benthic | 3 | N/A | S | Bahls, L. (2011). Navicula oblonga. In Diatoms of North America. Retrieved July 24, 2020, from https://diatoms.org/species/navicula_oblonga |
| *Navicula radiosa* | N/A | Benthic | 3 | N/A | All | Potapova, M. (2011). Navicula radiosa . In Diatoms of North America. Retrieved July 24, 2020, from https://diatoms.org/species/navicula_radiosa |
| *Navicula rhynchotella* | N/A | Benthic | N/A | N/A | S, L | Bahls, L. (2011). Navicula rhynchotella. In Diatoms of North America. Retrieved July 24, 2020, from https://diatoms.org/species/navicula_rhynchotella |
| *Navicula rostellata* | 46896 | Benthic | 5 | N/A | All | Potapova, M., Kociolek, P. (2011). Navicula rostellata . In Diatoms of North America. Retrieved July 24, 2020, from https://diatoms.org/species/navicula_rostellata |
| *Navicula symmetrica* | N/A | Benthic | N/A | D | All | Manoylov, K., Hamilton, P. (2010). Navicula symmetrica. In Diatoms of North America. Retrieved July 24, 2020, from https://diatoms.org/species/navicula_symmetrica |
| *Navicula tripunctata* | 46104 | N/A | 4 | N/A | All | Potapova, M. (2009). Navicula tripunctata. In Diatoms of North America. Retrieved July 24, 2020, from https://diatoms.org/species/navicula_tripunctata |
| *Navicula trivialis* | 46014 | Benthic | 4 | N/A | K, S, L | Rushforth, S., Spaulding, S. (2010). Navicula trivialis. In Diatoms of North America. Retrieved July 24, 2020, from https://diatoms.org/species/navicula_trivialis |
| *Navicula veneta* | 46504 | Benthic | 4 | N/A | All | Potapova, M. (2011). Navicula veneta . In Diatoms of North America. Retrieved July 24, 2020, from https://diatoms.org/species/navicula_veneta |
| *Nitzschia acidoclinata* | 48347 | Benthic | N/A | N/A | All | Bahls, L. (2016). Nitzschia acidoclinata. In Diatoms of North America. Retrieved July 24, 2020, from https://diatoms.org/species/nitzschia_acidoclinata |
| *Nitzschia amphibia* | 48004 | Benthic | 5 | D | All | Kociolek, P. (2011). Nitzschia amphibia. In Diatoms of North America. Retrieved July 24, 2020, from https://diatoms.org/species/nitzschia_amphibia |
| *Nitzschia cf microcephala* | 48024 | Benthic | 5 | N/A | All | Kociolek, P. (2011). Nitzschia microcephala. In Diatoms of North America. Retrieved July 24, 2020, from https://diatoms.org/species/nitzschia_microcephala |
| *Nitzschia cf sigma* | 48087 | Benthic | 4 | N/A | L | Kociolek, P. (2011). Nitzschia sigma. In Diatoms of North America. Retrieved July 24, 2020, from https://diatoms.org/species/nitzschia_sigma1 |
| *Nitzschia communis* | 48198 | Benthic | 5 | N/A | All | Kociolek, P. (2011). Nitzschia communis. In Diatoms of North America. Retrieved July 24, 2020, from https://diatoms.org/species/nitzschia_communis |
| *Nitzschia dissipata* | 48008 | Benthic | 3 | N/A | All | Manoylov, K. (2010). Nitzschia dissipata. In Diatoms of North America. Retrieved July 24, 2020, from https://diatoms.org/species/nitzschia_dissipata |
| *Nitzschia filiformis* | 48145 | Benthic | 4 | N/A | All | Kociolek, P. (2011). Nitzschia filiformis. In Diatoms of North America. Retrieved July 24, 2020, from https://diatoms.org/species/nitzschia_filiformis |
| *Nitzschia fonticola* | 48011 | Benthic | 4 | D | All | Kociolek, P. (2011). Nitzschia fonticola. In Diatoms of North America. Retrieved July 24, 2020, from https://diatoms.org/species/nitzschia_fonticola |
| *Nitzschia linearis* | 48023 | Benthic | 3 | N/A | All | Kociolek, P. (2011). Nitzschia linearis. In Diatoms of North America. Retrieved July 24, 2020, from https://diatoms.org/species/nitzschia_linearis |
| *Nitzschia palea* | 48025 | Benthic | 5 | D | All | Kociolek, P. (2011). Nitzschia palea. In Diatoms of North America. Retrieved July 24, 2020, from https://diatoms.org/species/nitzschia_palea |
| *Nitzschia paleacea* | 48165 | Benthic | 3 | N/A | All | Kociolek, P. (2011). Nitzschia paleacea. In Diatoms of North America. Retrieved July 24, 2020, from https://diatoms.org/species/nitzschia_paleacea |
| *Nitzschia recta* | 48029 | Benthic | 3 | N/A | S | Manoylov, K. (2010). Nitzschia recta. In Diatoms of North America. Retrieved July 24, 2020, from https://diatoms.org/species/nitzschia_recta |
| *Nitzschia sigmoidea* | 48177 | Benthic | 4 | N/A | All | Robson, S. (2017). Nitzschia sigmoidea. In Diatoms of North America. Retrieved July 24, 2020, from https://diatoms.org/species/nitzschia_sigmoidea |
| *Nitzschia soratensis* | N/A | Benthic | 2 | N/A | K, S, R | Stancheva, R. (2018). Nitzschia soratensis. In Diatoms of North America. Retrieved July 24, 2020, from https://diatoms.org/species/nitzschia_soratensis |
| *Pinnularia brebissonii* | 52018 | Benthic | N/A | N/A | All | Bahls, L. (2014). Pinnularia brebissonii. In Diatoms of North America. Retrieved July 24, 2020, from https://diatoms.org/species/pinnularia_brebissonii |
| *Planothidium frequentissimum* | 155017 | N/A | 4 | N/A | All | Potapova, M. (2010). Planothidium frequentissimum. In Diatoms of North America. Retrieved July 24, 2020, from https://diatoms.org/species/planothidium_frequentissimum |
| *Planothidium lanceolatum* | 155003 | N/A | 4 | C | All | Potapova, M. (2010). Planothidium lanceolatum. In Diatoms of North America. Retrieved July 24, 2020, from https://diatoms.org/species/planothidium_lanceolatum |
| *Reimeria sinuata* | 55002 | Benthic | 2 | N/A | All | Potapova, M. (2009). Reimeria sinuata. In Diatoms of North America. Retrieved July 24, 2020, from https://diatoms.org/species/reimeria_sinuata |
| *Rhoicosphenia abbreviata* | 57002 | Benthic | 3 | N/A | All | Potapova, M. (2009). Rhoicosphenia abbreviata. In Diatoms of North America. Retrieved July 24, 2020, from https://diatoms.org/species/rhoicosphenia_abbreviata |
| *Sellaphora laevissima* | 170001 | Benthic | 3 | N/A | S, L | Burge, D., Edlund, M. (2017). Sellaphora laevissima. In Diatoms of North America. Retrieved July 24, 2020, from https://diatoms.org/species/sellaphora_laevissima |
| *Stauroneis anceps* | 62002 | Benthic | 3 | N/A | S | Bahls, L. (2011). Stauroneis anceps. In Diatoms of North America. Retrieved July 24, 2020, from https://diatoms.org/species/stauroneis_anceps |
| *Staurosira construens* | 172001 | Benthic/Planktonic | 4 | N/A | K, S, L, R | Morales, E. (2010). Staurosira construens. In Diatoms of North America. Retrieved July 24, 2020, from https://diatoms.org/species/staurosira_construens |
| *Stephanodiscus minutulus* | 64018 | Planktonic | N/A | N/A | K, S, L | Burge, D., Edlund, M. (2017). Stephanodiscus minutulus. In Diatoms of North America. Retrieved July 24, 2020, from https://diatoms.org/species/stephanodiscus_minutulus |
| *Surirella brebissonii* | 65068 | Benthic | 5 | N/A | All | English, J., Potapova, M. (2011). Surirella brebissonii. In Diatoms of North America. Retrieved July 24, 2020, from https://diatoms.org/species/surirella_brebissonii |
| *Surirella ovalis* | 65043 | Benthic | 5 | N/A | All | English, J. (2011). Surirella ovalis. In Diatoms of North America. Retrieved July 24, 2020, from https://diatoms.org/species/surirella_ovalis |
| *Tryblionella apiculata* | 185023 | Benthic | 5 | N/A | All | Penton, M. (2013). Tryblionella apiculata. In Diatoms of North America. Retrieved July 24, 2020, from https://diatoms.org/species/tryblionella_apiculata |
| *Ulnaria acus* | 245005 | Benthic/Planktonic | N/A | N/A | All | Burge, D., Tunno, I., Edlund, M. (2016). Ulnaria acus. In Diatoms of North America. Retrieved July 24, 2020, from https://diatoms.org/species/ulnaria_acus |

*NADED ID: Diatoms of North America database identification number

**BCG (Biological Condition Gradient): 1 = specialist species, 2 = highly sensitive species, 3 = sensitive species, 4 = indiscriminate species and 5 = tolerant species;

*** Eastern Canadian Diatom Index: Class A = Reference Conditions, Class B = Slightly Altered, Class C = Altered, Class D = Severely Altered Conditions

+Detection: K = kick-net, S = sediment, L = leaf litter, M = macrophyte, R = rock scraping, All = detected in all sampling methods.

N/A: Information not available

**
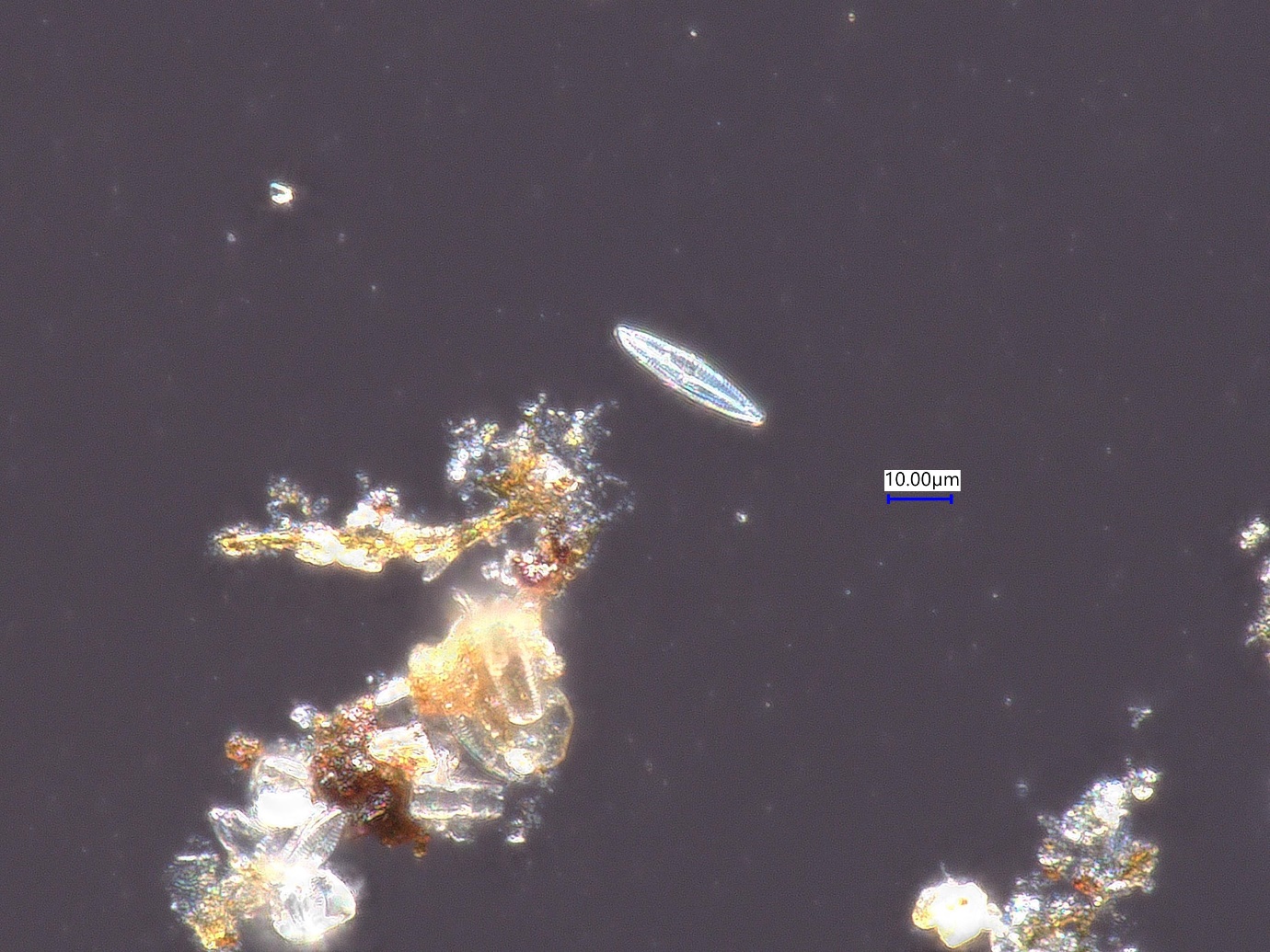
**

**S1 Fig. Example of confirmation of diatom presence from preservative of kick-net sample.** Image: CBG Photography Group.


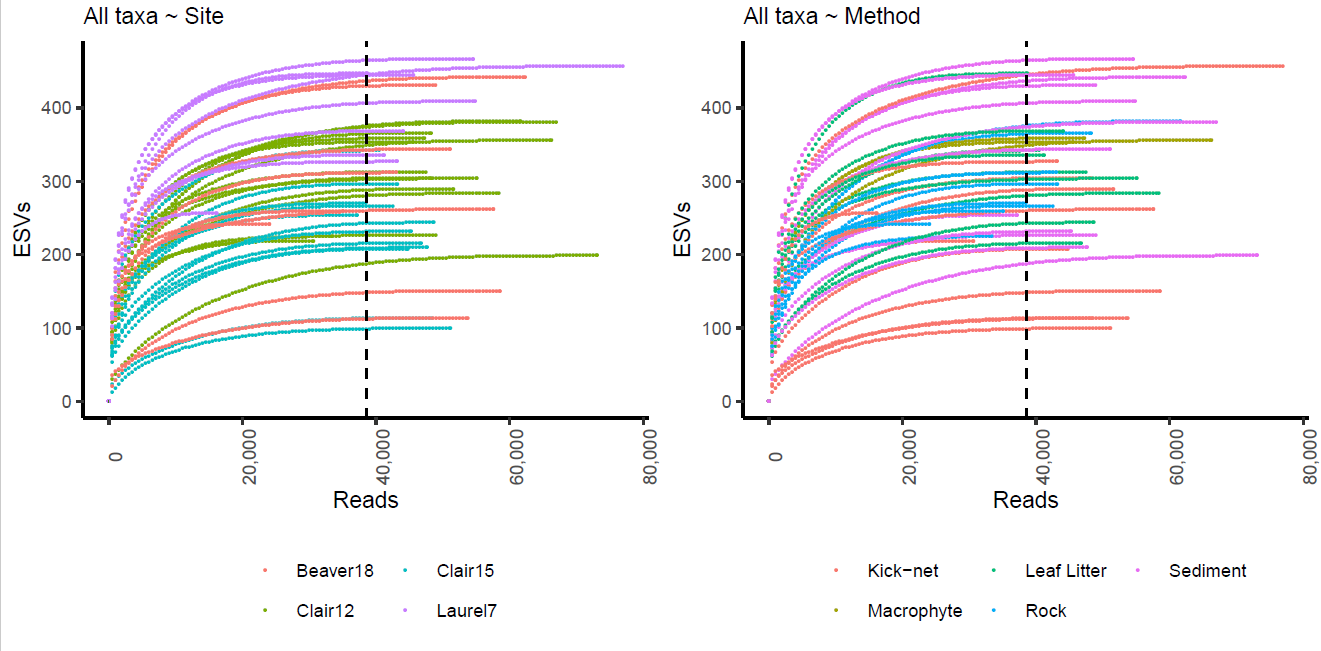


**S2 Fig. All samples show that ESV sampling reached saturation.** Each line represents reads from a sample plotted against the number of detected ESVs. Samples were color-coded by site or method as shown in the legend. The vertical dashed line indicates the 15th percentile of sampling read depth, which is the number of reads that would be used in any future analysis based on normalized data.


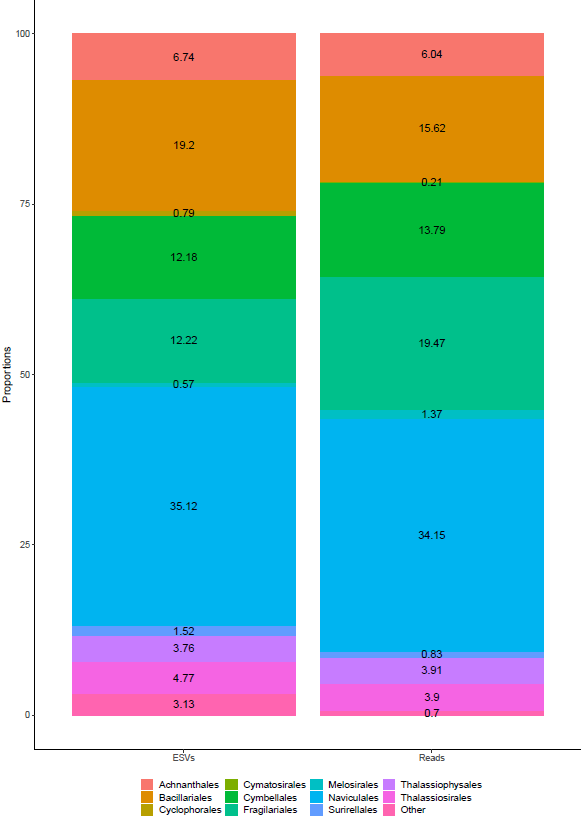


**S3 Fig. *Naviculales* is the most abundant diatom order detected.** Results for the top 10 orders are shown with respect to proportion of ESVs and reads recovered. Based on raw unnormalized data.


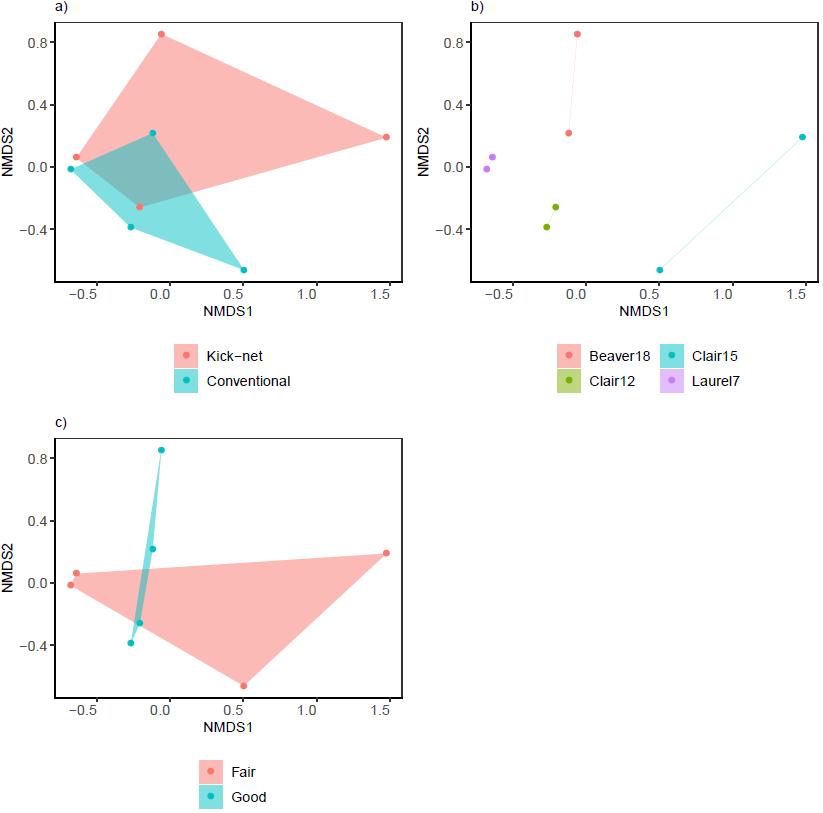


**S4 Fig.** **Non-metric multi-dimensional scaling plots of microhabitat samples pooled show clustering by due to site and status**. Specifically, a) depicts overlap between the binary Bray Curtis (Sorensen) dissimilarities between different sampling approaches, b) sample site clustering c) clustering based on habitat quality status. (stress = 0.043, R^2^ = 0.99). Based on rarefied data.


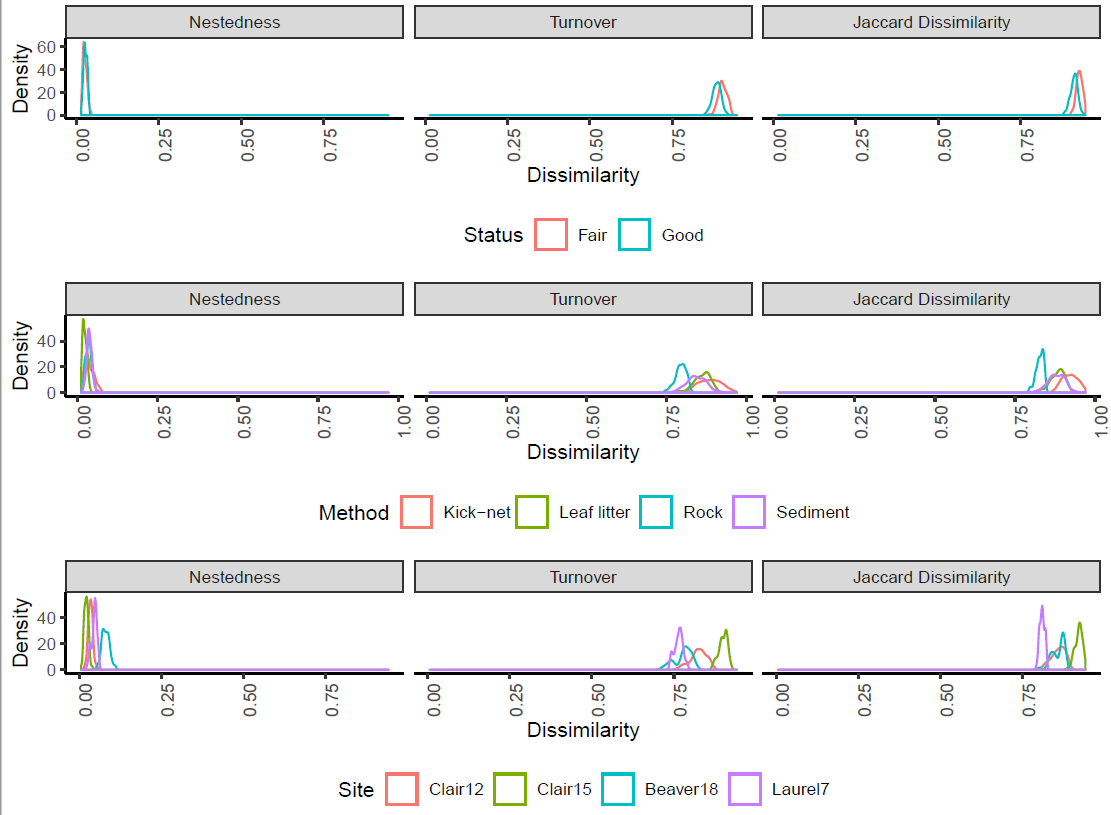


**S5 Fig. Diatom nestedness, turnover and Jaccard dissimilarity.** Top panel: samples pooled across methods and sites; middle panel: samples pooled across site status and site; bottom panel: samples pooled across method and site status.
